# Performance assessment of a triple-junction solar cell with 1.0 eV GaAsBi absorber

**DOI:** 10.1186/s11671-023-03865-x

**Published:** 2023-06-16

**Authors:** Tadas Paulauskas, Vaidas Pačebutas, Viktorija Strazdienė, Andrejus Geižutis, Jan Devenson, Mindaugas Kamarauskas, Martynas Skapas, Rokas Kondrotas, Mantas Drazdys, Matas Rudzikas, Benjaminas Šebeka, Viliam Vretenár, Arūnas Krotkus

**Affiliations:** 1 Center for Physical Sciences and Technology, Saulėtekio Ave. 3, 10257 Vilnius, Lithuania; 2 Applied Research Institute for Prospective Technologies, Vismaliukų St. 34, 10243 Vilnius, Lithuania; 3 Center for Nanodiagnostics of Materials, Faculty of Materials Science and Technology, Slovak University of Technology, Vazovova 5, 812 43 Bratislava, Slovakia

**Keywords:** Multi-junction solar cells, III–V photovoltaics, GaAsBi, Molecular beam epitaxy

## Abstract

Group III–V semiconductor multi-junction solar cells are widely used in concentrated-sun and space photovoltaic applications due to their unsurpassed power conversion efficiency and radiation hardness. To further increase the efficiency, new device architectures rely on better bandgap combinations over the mature GaInP/InGaAs/Ge technology, with Ge preferably replaced by a 1.0 eV subcell. Herein, we present a thin-film triple-junction solar cell AlGaAs/GaAs/GaAsBi with 1.0 eV dilute bismide. A compositionally step-graded InGaAs buffer layer is used to integrate high crystalline quality GaAsBi absorber. The solar cells, grown by molecular-beam epitaxy, achieve 19.1% efficiency at AM1.5G spectrum, 2.51 V open-circuit voltage, and 9.86 mA/cm^2^ short-circuit current density. Device analysis identifies several routes to significantly improve the performance of the GaAsBi subcell and of the overall solar cell. This study is the first to report on multi-junctions incorporating GaAsBi and is an addition to the research on the use of bismuth-containing III–V alloys in photonic device applications.

## Introduction

Space and terrestrial concentrated-sun photovoltaics are the primary applications of multi-junction (MJ) solar cells [1, 2]. In both operating conditions, the highest achievable efficiency is determined by bandgap combinations of its constituent subcells. However, in practice, the efficiency of MJ devices is also determined by their materials aspect. Given the scalability and stringent application requirements, any new MJ architecture must prove itself against the mature GaInP/InGaAs/Ge triple-junction (3 J) technology [3].

Emerging multi-junction solar cells hold the promise of achieving higher efficiency than the current commercial 3 J Ge-based cells, which currently achieve 30% under the AM0 spectrum. The most common approach is to retain the top two high-quality lattice-matched subcells, namely the GaInP (1.8–1.9 eV bandgap) and the InGaAs (1.35–1.41 eV) subcells, while increasing the bandgap of the bottom subcell to 0.9–1.0 eV [4]. To date, the best performance for the bottom subcell has been demonstrated by the InGaAs alloy [3–7]. Several versions of such 3 J solar cells have been proposed, with the best achieving 39.5% efficiency under one-sun AM1.5G and 34.2% under AM0 space spectra [8]. The design of 3 J devices that incorporate bottom InGaAs subcell relies on a several micrometres thick buffer layer and an inverted metamorphic (IMM) epitaxy to accommodate an almost 2% lattice mismatch [5, 9, 10]. Dilute nitrogen-based alloys, such as GaInNAs and GaInNAsSb, have also shown potential for use in 3 J and 4 J devices because they allow for bandgap variation in the 0.7–1.4 eV range while maintaining the desired lattice constant [11, 12]. A triple-junction cell incorporating GaInNAsSb has demonstrated an efficiency of 44.4% at a concentrated-sun AM1.5G spectrum. However, despite their potential, nitrogen-based alloys have not gained wider adoption in photovoltaics yet due to voltage-limiting factors that require further attention.

Dilute bismide GaAs_1−*x*_Bi_*x*_ is similar to dilute nitrides in that both demonstrate large bandgap reduction at low isoelectronic dopant concentrations. With GaAsBi, the bandgap reduction of as much as 80–90 meV/Bi% can be achieved, and at 1.0 eV bandgap, its lattice mismatch with GaAs is only 0.6% [13, 14]. Incorporation of Bi mainly perturbs the valence band, unlike dilute nitrogen alloys in which the conduction band is affected more. This difference offers alternative electronic band engineering methods in photovoltaics. Furthermore, much thinner and less complicated buffer layer is needed to incorporate 1.0 eV GaAsBi in an MJ device, as compared to InGaAs, due to a smaller lattice mismatch [15]. However, unlike dilute nitrogen alloys, the growth mechanisms and materials properties of GaAsBi alloy are less explored. For example, only recently it was shown that typical dilute GaAsBi layers grown on (001) GaAs possess pronounced optical anisotropy associated with the ubiquitous CuPt_B_-type ordering [16–18]. Little work has been done on growing high crystal quality thick GaAsBi layers that are needed in solar cell applications [19–21]. This is likely due to the metastability of GaAsBi, which requires temperatures below 400 °C and fine-tuning of III/V elemental ratios to incorporate Bi into GaAs lattice [13, 22]. Recent works on molecular-beam epitaxy (MBE) growth of GaAsBi on lattice-matched InGaAs buffers suggest that crystal quality and Bi solubility can be enhanced [15, 23–25]. In our recent study, the growth and analysis of GaAsBi/InGaAs heterojunction 1.0 eV solar cells was reported, where we improved on the previous GaAsBi photovoltaic results [26]. Nevertheless, there have been limited reports so far on the use of GaAsBi in solar cell applications. Studies have mainly focused on the material properties or use of bismide as a single-junction absorber.

This work is the extension of the GaAsBi material applications into multi-junction solar cells. We present an MBE-grown thin-film triple-junction AlGaAs/GaAs/GaAsBi solar cell with a 1.87/1.42/1.00 eV bandgap combination. The performance of the solar cells was evaluated through current–voltage measurements under AM1.5G illumination, external quantum efficiency measurements, and device modelling.

## Experimental methods

The multi-junction solar cell was grown in a Veeco GENxplor MBE chamber equipped with solid-state sources. A valved cracker was used for the As source. Doping was performed with Be, Si, and Te. The latter was switched on only at the tunnel junction regions to supplement Si doping. The cell was grown on a quarter of a 2″ semi-insulating (001) GaAs epi-ready substrate (Wafertech, Ltd). The substrate temperature was controlled by a thermocouple and monitored with a kSA BandIt broadband pyrometry module. The outgassing and oxide removal from the substrate were performed at standard temperatures. The layers were grown at 0.6–0.7 µm/h, except the tunnel junctions, which were grown at 0.1 µm/h speed. The growth temperature of all layers up to the step-graded InGaAs buffer was 580 °C. The InGaAs buffer was deposited at 500 °C. The InGaAs buffer and GaAsBi layer were grown using optimized parameters described elsewhere in [15]. The back contact InGaAs layers were deposited at 430 °C.

A thin-film triple-junction solar cell was obtained by GaAs substrate etching. The etching procedure, including back surface metallization with metal contacts, electrochemical deposition of a 20-μm-thick Cu, and securing the cell to a polyimide sheet, is described elsewhere in [10].

The top contact was formed using standard photolithography procedures. The metal lift-off procedure was carried out in acetone at room temperature. Trenches between the active areas and edges of the sample were etched with a mixture of phosphoric acid and hydrogen peroxide. AZ1518 (MicroChemicals) photoresist was employed as a protective coating layer for the active area during etching.

Veeco Savannah S200 atomic layer deposition (ALD) reactor, equipped with a capacitively coupled plasma generator, was used to deposit antireflective coating TiO_2_/SiO_2_ (58.3/114.0 nm) at 60 ℃ substrate temperature. Before the deposition, the GaAs contact layer was etched down to the AlGaAs window layer using a selective etching solution NH_4_OH (28%): H_2_O_2_ (35%): H_2_O = (1:4:20). The samples were then immediately placed in a vacuum desiccator. The ARC was deposited using tetrakis(dimethylamino)titanium and tris(dimethylamino)silane as precursors using oxygen plasma as an oxygen source.

The *J*–*V* characteristics were measured at room temperature on a Keithley 4200-SCS (Tektronix) semiconductor parameter analyser. A fully reflective solar simulator SS1.6 K (ScienceTech) was used to illuminate samples with AM1.5G spectral light. High-voltage Xenon lamp was used as a light source. External quantum efficiency (EQE) measurements were performed with a Bentham PVE300 spectrometer. Wavelength was varied from 300 to 1400 nm with a step size of 5 nm, and the mixture of halogen and xenon lamps was used as a light source with the monochromator. An additional halogen lamp was used for light bias with the following filters from Schott: RG630 (for the top junction), BG23 (for the middle junction), and KG1 (for the middle junction). For the middle and bottom junctions, an infrared mirror was placed next to the other filters. A voltage bias was set when measuring the bottom cell, as described in [27].

Calculation of the solar cell’s band edge diagram was performed with the Silvaco TCAD software suite. Materials parameters for the GaAsBi/InGaAs subcell used in the simulation are described elsewhere in [26].

Structural and elemental analysis of the multi-layered structure was carried out using a cold-field-emission aberration-corrected scanning transmission electron microscope (STEM) JEM-ARM 200cF (JEOL) operated at 200 kV, equipped with a 100 mm^2^ silicon drift-detector JED-2300 (JEOL) for energy-dispersive spectroscopy imaging (EDS). For STEM imaging, the microscope’s parameters were set as follows: probe convergence semi-angle of 22 mrad; high-angle annular dark-field (HAADF) detector’s inner and outer semi-angles of 68 and 280 mrad, respectively; low-angle annular dark-field (LAADF) detector’s inner and outer semi-angles of 30 and 120 mrad, respectively; and bright-field (BF) detector’s outer semi-angles of 34 mrad. High-resolution phase-contrast TEM imaging was carried out using FEI Tecnai G2 F20 X-TWIN operated at 200 kV accelerating voltage. Cross-sectional samples were prepared in a focused ion beam (FIB) FEI Helios Nanolab 650 microscope using the lift-off technique.

## Results and discussion

### Device structure

**Fig. 1:**
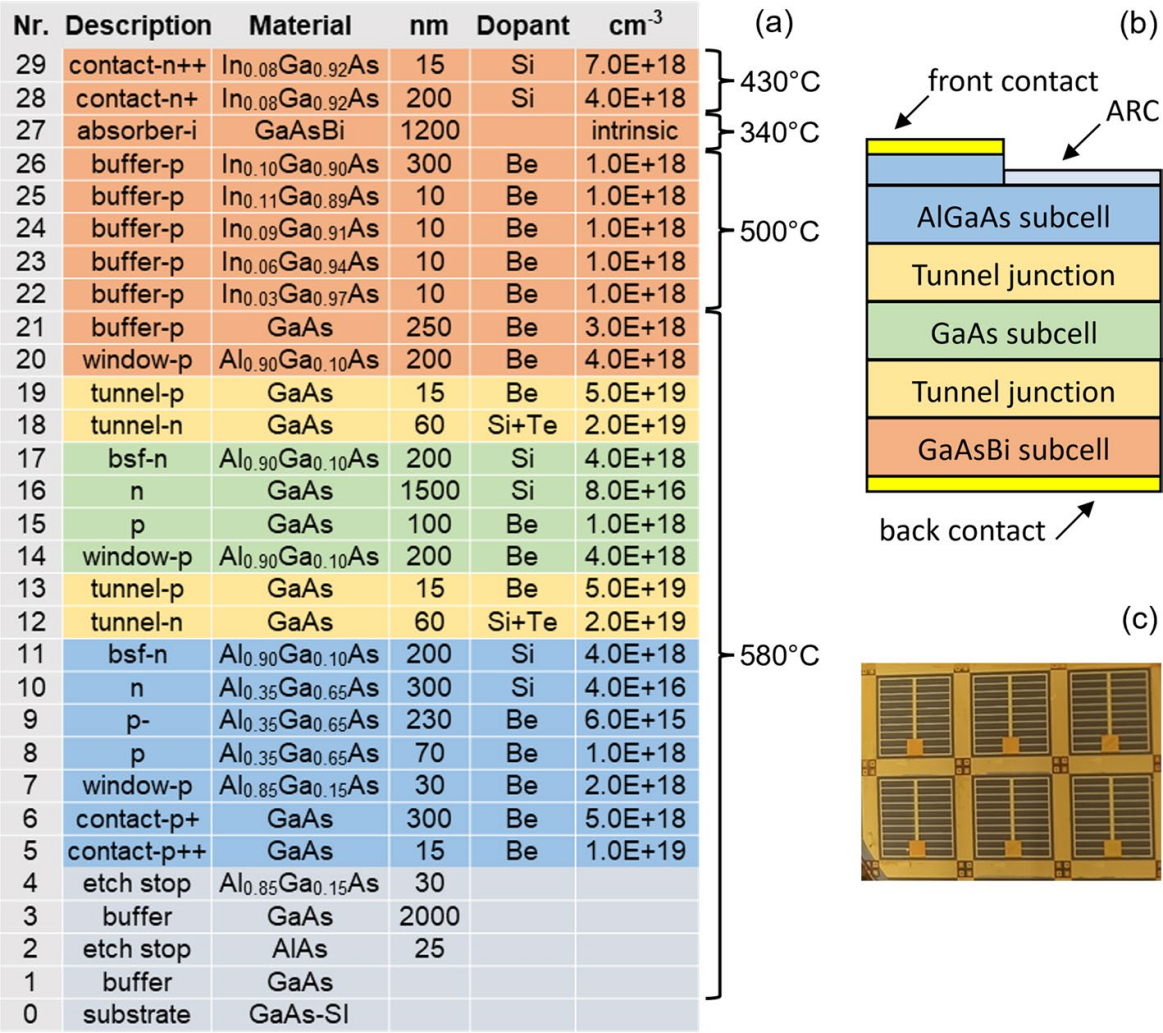
**a** Layer structure of the triple-junction solar cell grown on SI-GaAs substrate. It shows used alloys and their description, layer thicknesses (nm), dopant atoms, and carrier concentrations (cm^−3^). BSF abbreviates back surface field. Substrate temperatures (as measured by BandIt pyrometer) at which the growth of different parts of the solar cell was performed are shown on the right. **b** Depiction of a completed solar cell with removed GaAs substrate. **c** Photograph of completed solar cell mesa structures with ARC and contact grids. The active area of each mesa solar cell, considering grid shading, measures to 0.2 cm^2^

The triple-junction solar cell featuring a GaAsBi absorber layer is depicted in Fig. 1a, b. The solar cell is grown inverted on a semi-insulating GaAs substrate starting from the top highest bandgap Al_0.35_Ga_0.65_As subcell, followed by the GaAs middle subcell, and finally, the GaAsBi-based bottom subcell. After growth, a back contact of AuGe/Ni/Au is deposited, followed by electrochemical copper deposition and attachment of the structure to a Kapton polyimide. The substrate is then removed by etching [10]. Layers 2–4 (see Fig. 1a) are employed to assist with the substrate etching steps and further device processing. Standard photolithography steps are carried out to form top contacts. A thin-film 3 J solar cell, obtained through the aforementioned process, is illustrated in Fig. 1b, while Fig. 1c shows a photograph of a completed device with several mesa cells.

The decision to use the inverted growth method was primarily driven by two factors. Firstly, the instability of the GaAsBi alloy above around 450 °C leads to the ejection of Bi from the lattice and formation of Bi droplets [13, 28]. The higher substrate temperatures required to grow the AlGaAs and GaAs subcells (580 °C, see Fig. 1a) with good crystal quality would damage the GaAsBi subcell, making it necessary to grow the bismide layer last. Secondly, an InGaAs buffer layer was utilized to increase the lattice constant to match that of the bismide. The Al_*x*_Ga_1-*x*_As alloy is almost exactly lattice-matched with GaAs for all *x*, for the practical purposes at hand. Therefore, by growing the top two subcells first, the likelihood of their crystal structure being compromised by dislocations generated in the InGaAs buffer layer is significantly reduced. We acknowledge that alternative material choices for the graded buffer layer, instead of InGaAs, could be used [5, 7, 8]. In fact, using a buffer layer material with a larger bandgap, such as GaInP, GaAsP, or AlInGaAs, would be advantageous in reducing parasitic absorption. In this case, InGaAs was chosen due to the availability of sources and a preference for its lower growth temperature to maintain the stability of the tunnel diodes.

The *p*-on-*n* device polarity, utilized in this 3 J device, was largely determined by the Be dopant diffusion properties in the tunnel diodes. Previous studies, along with our secondary-ion mass spectroscopy studies (not shown here), have demonstrated that Be diffuses towards the growth surface, making it challenging to maintain high doping density (on the order of 10^19^ cm^−3^) in a thin region [29, 30]. Furthermore, the diffused Be may also compensate the *n*-type doped tunnel diode region if the *p*-type tunnel portion is grown first. We found that using an *n*-on-*p* tunnel diode polarity, coupled with 200-nm-thick Al_0.90_Ga_0.10_As barrier layers encompassing the tunnel diodes, allows for retaining their stability and peak tunnelling current densities 20–30 mA/cm^2^ under prolonged annealing times experienced by the tunnel diode during the full device growth period [31]. For the *n*-type tunnel diode regions, we employed a co-doping of Si with Te to enhance dopant stability and raise carrier density to 2 × 10^19^ cm^−3^.

Studies have shown that an intrinsic 1.0 eV GaAsBi (~ 5.5% Bi) is slightly *p*-type with a carrier density of 1 × 10^15^–1 × 10^16^ cm^−3^ [32]. Although it is possible to dope GaAsBi either *p*- or *n*-type, which could potentially improve the subcell’s *V*_oc_, there is a risk that already low carrier lifetimes in the bismide will decrease even further [26, 33]. To stay on the safe side, we used an intrinsic GaAsBi. For the intrinsic GaAsBi subcell, the *p*-on-*n* device polarity is more favourable over the *n*-on-*p* because it provides an electric field at the back of the absorber, which enhances photogenerated carrier extraction.

**Fig. 2:**
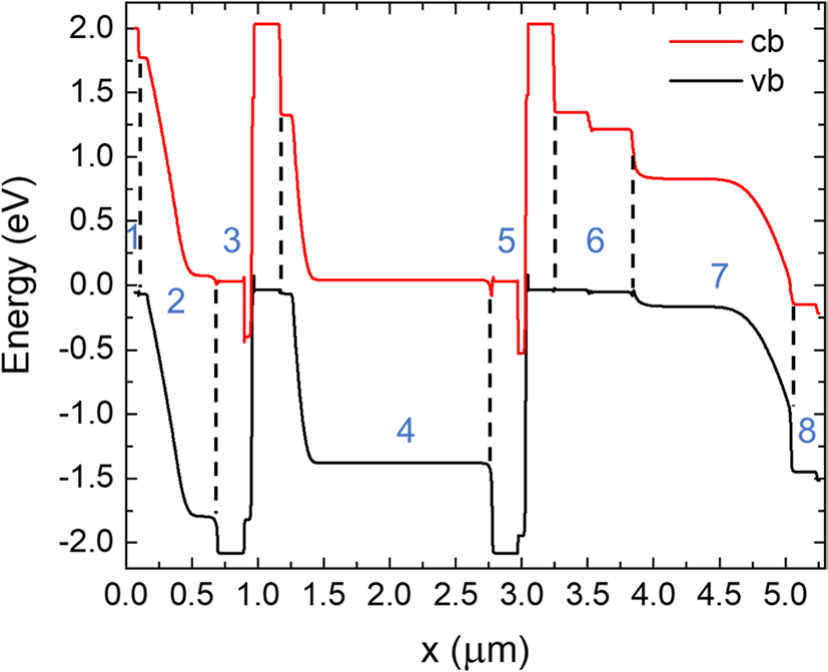
Conduction (cb) and valence band (vb) edges at different positions in the GaAsBi-based triple-junction solar cell. The Fermi level is at 0.0 eV, and simulation was done in thermal equilibrium conditions. The numbering indicates: 1—top window layer, 2—AlGaAs subcell, 3—first tunnel diode, 4—GaAs subcell, 5—second tunnel diode, 6—GaAs/InGaAs buffer layer, 7—GaAsBi absorber layer, 8—InGaAs back contact layer

The triple-junction solar cell was modelled using the Silvaco TCAD software suite. Figure 2 shows the diagram of the conduction and valence band edges. It should be noted that the actual band bending may differ somewhat due to dopant diffusion, particularly in the tunnel diode regions. As can be seen in Fig. 2, the electric field extends over most of the AlGaAs top subcell, which facilitates charge carrier extraction. Regarding the bismide absorber, the band bending is situated towards the back and so quantum efficiency at longer wavelength photons should be enhanced in the *p*-*i*-*n* bismide configuration. We note that the *p*- and *n*-layers of the bismide diode are InGaAs alloys, thereby formally forming a heterojunction diode. The figure also indicates that no significant electronic barriers for charge carrier extraction should exist at the InGaAs/GaAsBi interfaces.

The materials parameters for the modelling of the bismide were adopted from our recent article on GaAsBi/InGaAs solar cells as the growth conditions for GaAsBi in the 3 J device were kept the same [26]. The simulations used 0.2 ns electron and 0.05 ns hole lifetimes, as well as 1800 cm^2^/V s and 20 cm^2^/V s mobilities, respectively. With these parameters, the bismide subcell limits the short-circuit current of the 3 J device and the subcells were thinned accordingly. The simulations suggest that a short-circuit current density (*J*_sc_) of 12.2 mA/cm^2^ can be achieved under the AM1.5G spectrum. The modelled device exhibited a *V*_oc_ of 2.57 V, fill factor (*FF*) of 0.78, and power conversion efficiency (*η*) of 24.5%.

**Fig. 3:**
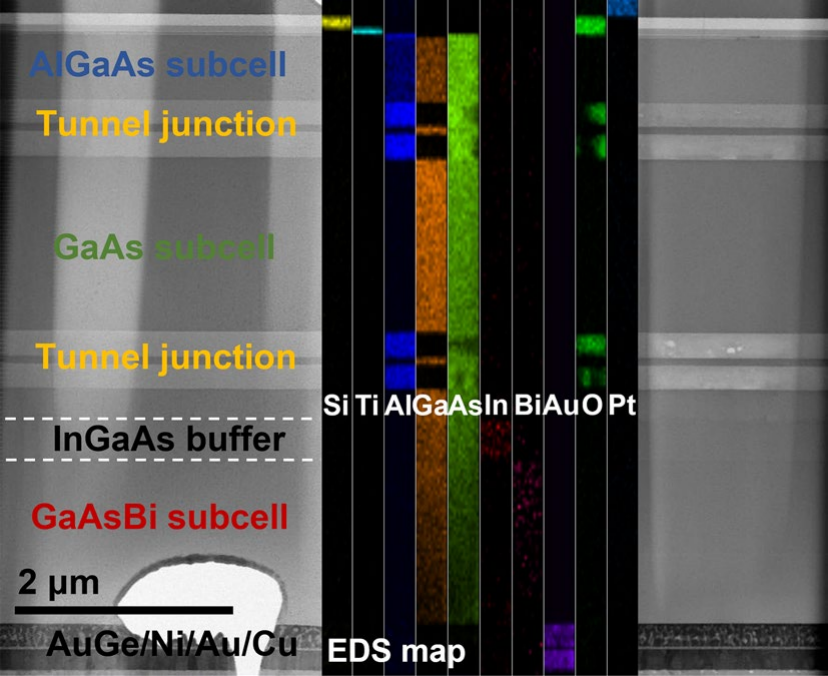
BF-STEM and EDS characterization of the cross-sectional 3 J device along [110] zone-axis. BF-STEM image shows the entire triple-junction solar cell. Subcells and different layers are labelled, including the position of the step-graded InGaAs buffer layer. The inset, overlaid on the STEM image, displays EDS elemental map of a narrow sample region

To investigate the structural quality of the triple-junction solar cell, cross-sectional scanning transmission electron microscopy (STEM) characterization was carried out. Figure 3 displays a bright-field (BF) STEM image of the solar cell, with an energy-dispersive spectroscopy (EDS) imaging inset. The sample surface was covered with Pt to protect it during focused ion beam (FIB) sample preparation. Below the Pt, the double ARC layer, different subcells, GaAs tunnel junctions, and back contact metals are visible. The thin tunnelling region is surrounded by 200-nm-thick visibly brighter AlGaAs layers (layers 11, 14, 17, and 20 in Fig. 1a). The exposed high Al content AlGaAs layers readily oxidize in air, and this can be seen in the EDS map. The vertical columnar-like contrast is due to TEM sample thickness variations, and the void in GaAsBi subcell is from FIB damage. The sample becomes thinner towards the back contact, which is apparent from the diminishing Ga and As EDS signals. A weak bismuth EDS signal can be seen from the dilute GaAsBi absorber layer. We note that no phase-separation or Bi droplets were detected in the 1200-nm-thick GaAsBi layer, as determined by STEM imaging. This confirms optimized growth conditions for high structural quality GaAsBi employed in these devices.

**Fig. 4:**
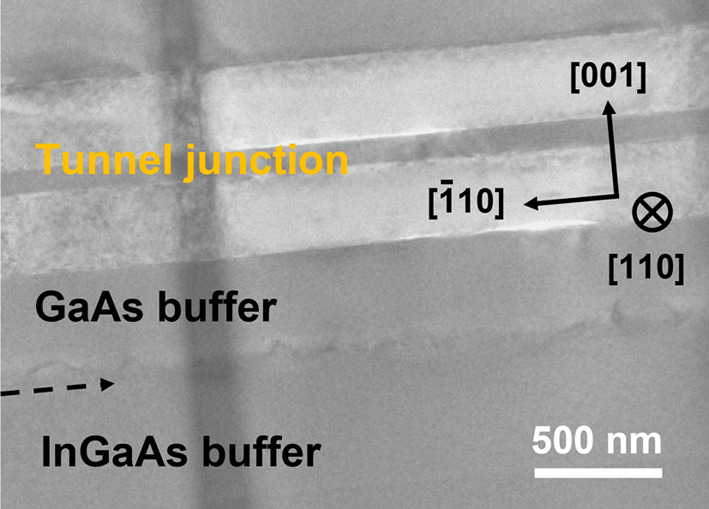
BF-TEM image of the sample near the InGaAs buffer layer. The visible corrugated contrast at the GaAs/InGaAs interface, marked with an arrow, is due to dislocations. Crystallographic directions of the sample are indicated

A zoomed-in BF-TEM image of the InGaAs buffer layer region (layers 22–26) is shown in Fig. 4. The corrugated contrast visible along the GaAs buffer (layer 21) and InGaAs buffer interface is attributed to the high density of dislocations. Notably, no significant dislocation contrast is visible in the GaAsBi absorber layer (Fig. 4). In our previous study, we reported an upper limit for the dislocation density, *ρ*, in the GaAsBi layer grown on a step-graded InGaAs buffer configuration, which was determined to be *ρ* < 1 × 10^8^ cm^−2^ [15]. The dislocations were primarily concentrated at the GaAs/InGaAs interface and within the four 10-nm-thick InGaAs buffer layers, similar to our observations in the current device. In future studies, additional techniques such as cathodoluminescence or surface etch pit counting could be employed to further refine the assessment of dislocation density.

### Photovoltaic performance

**Fig. 5:**
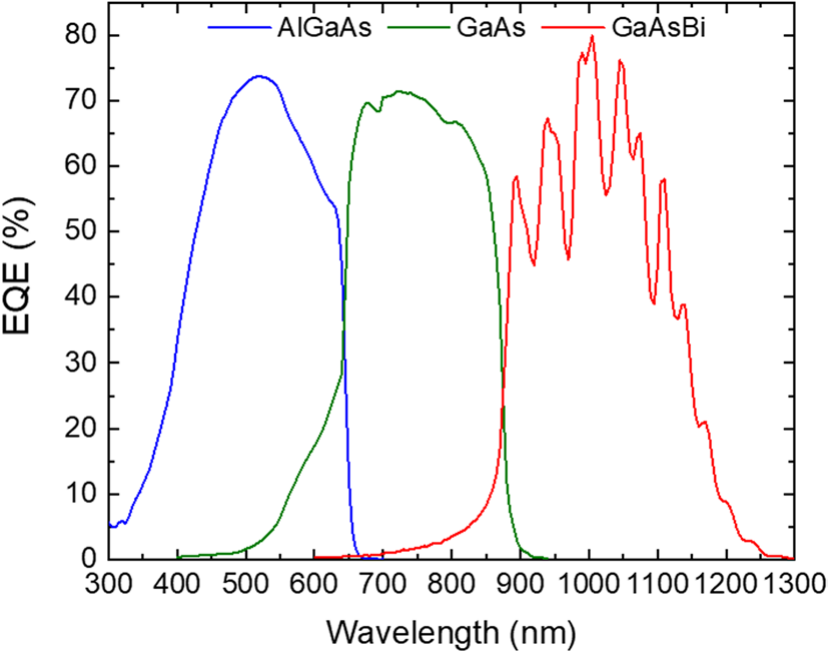
External quantum efficiency of the 3-junction solar cell

**Fig. 6:**
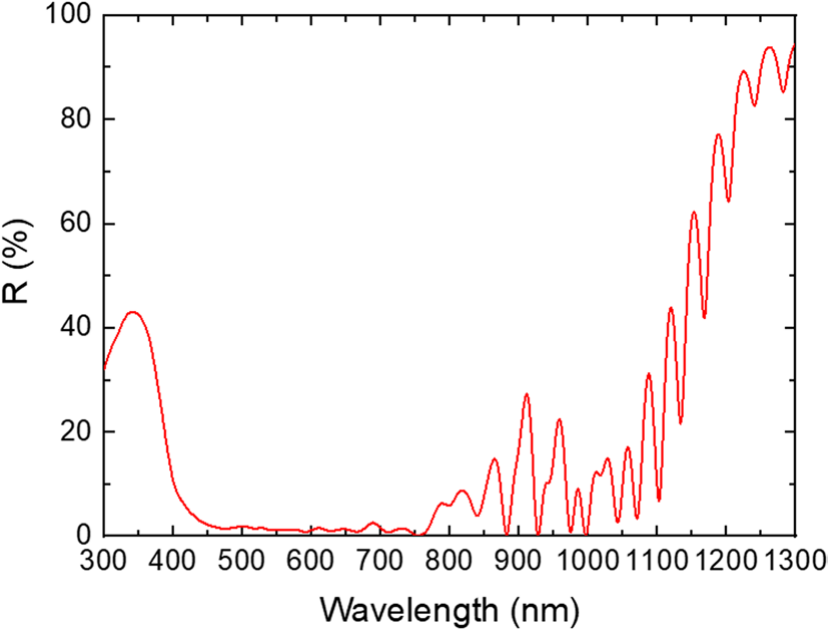
Reflectance spectrum of the 3-junction obtained using device simulation

Figure 5 shows the EQE results of a representative solar cell. The spectral response of the GaAsBi subcell extends up to 1250 nm wavelength and slightly beyond. Simulations suggest that the reflectance in the 1000–1250 nm spectral range increases from 5 to 90% (refer to Fig. 6). The tunnel diode regions, encapsulated by the Al_0.90_Ga_0.10_As layers, strongly reflect longer wavelength photons that should otherwise reach the bottom GaAsBi subcell. The Fabry–Perot interference effects are also visible due to the reflection of light from the back metal contact and internal reflections in the solar cell. The reflection from the back contact effectively increases the optical path in GaAsBi allowing for a thinner absorber. To improve carrier extraction at the top AlGaAs subcell, graded doping levels and a reduced *n*-type AlGaAs base thickness were employed [34]. Due to thinned AlGaAs layer used for current matching and resultant incomplete absorbance, spectral overlap with GaAs subcell in the 500–650 nm region can be seen in Fig. 5.

**Fig. 7:**
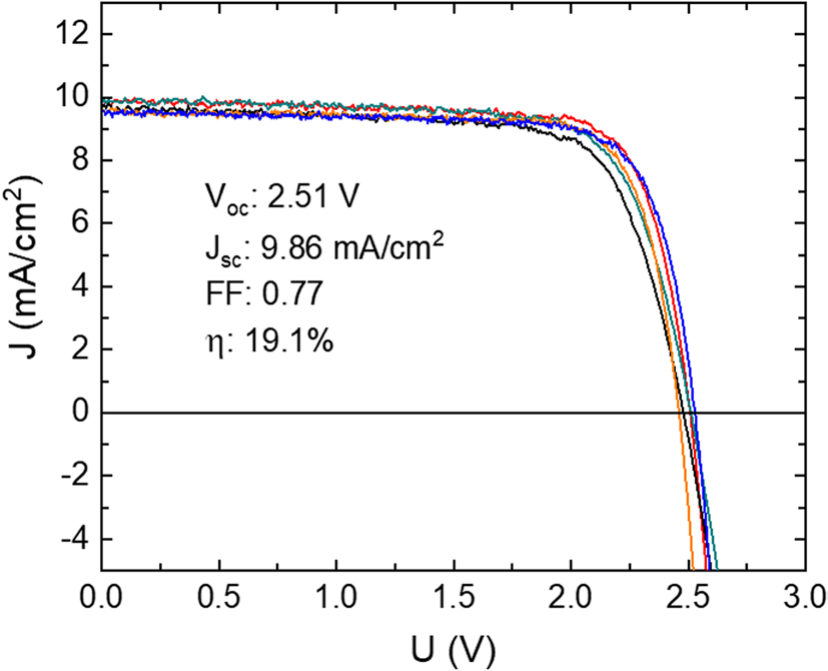
Room-temperature *J*–*V* characteristics of five 3-junction solar cell mesa structures obtained under AM1.5G solar spectrum. The inset values refer to the best efficiency solar cell in this sample set

The *J*–*V* data of the triple-junction solar cell are presented in Fig. 7, acquired at room temperature under AM1.5G solar spectrum illumination. The inset in Fig. 7 lists the photovoltaic characteristics of the most efficient cell (*η* = 19.1%, red curve) in the set. The *V*_oc_ of the solar cells varies from 2.46 to 2.53 V, *J*_sc_ ranges from 9.50 to 9.90 mA/cm^2^, and FF ranges from 0.72 to 0.78. The spread in *V*_oc_ and FF values could be attributed to several factors, including sample processing effects and spatial elemental inhomogeneities that may have occurred during growth. Our investigations show that the *V*_oc_ of a standalone AlGaAs top cell can vary between 1.20 and 1.25 V, *V*_oc_ of a GaAs cell—between 0.98 and 1.0 V, while a 0.99 eV bandgap GaAsBi demonstrated 0.34 V in our previous study [26]. Based on these values, we can expect the *V*_oc_ of the triple-junction solar cell to be in the range of 2.52–2.59 V.

Next, we will discuss strategies for improving the performance of the 3 J solar cell. Firstly, it is important to note that the overall architecture of the 3 J device was partially influenced by the available sources and dopants in the MBE system. We utilized an Al_*x*_Ga_1-*x*_As alloy for the top subcell, which is known to have suboptimal performance characteristics. High concentrations of Al can result in reduced carrier lifetimes and a significant bandgap-voltage offset (*W*_oc_ = *E*_g_/*q* − *V*_oc_), potentially attributed to the presence of oxygen-related or DX-defect centres [35, 36]. In addition, there may be a high interface recombination velocity at the AlGaAs window and back surface field layers, which can diminish the EQE of the subcells. By substituting the top AlGaAs subcell with GaInP, we anticipate an increase in *V*_oc_ by up to 0.3 V, resulting in a 21% efficiency for the 3 J device (using the relation *η* = *V*_oc_ × *J*_sc_ × *FF*/*P*), assuming all other factors remain constant. Furthermore, reducing the thickness of the tunnel diode encapsulation layers and replacing them with phosphorus-based alloys such as AlInP, GaInP, or AlGaInP could mitigate long-wavelength reflection losses and enhance the EQE of the GaAsBi subcell. We estimate that implementing these changes alone could achieve a current-matching *J*_sc_ of approximately 12.0 mA/cm^2^, and utilizing the GaInP top subcell would yield an efficiency of 26%. Further efficiency improvements of up to 2 percentage points are possible by increasing the fill factor to 0.82–0.84. It is reasonable to expect that the *FF* would be improved to some extent due to the aforementioned material choices. As for the GaAs subcell, the growth of GaAs crystals is well developed and near-optimal cell performance with a *V*_oc_ of 1.0 V can be routinely achieved.

Several approaches can be considered to improve the performance of the GaAsBi subcell in the MJ device. As mentioned previously, the reflection losses that affect EQE could be reduced through light management and alternative materials to AlGaAs. Another method to increase subcell current is to use a patterned back contact layer and a thicker GaAsBi absorber. Our previous modelling suggests that the optimal thickness is around 1400 nm [26]. Voltage offsets, *W*_oc_, in GaAsBi are typically 0.60–0.65 V and tend to decrease with decreasing Bi concentration [19, 22]. Short charge carrier lifetimes, on the order of 0.1 ns, seem to be the primary cause of large *W*_oc_ values in GaAsBi, which also negatively affects the *J*_sc_ [26]. Currently, the lifetimes in GaAsBi may be influenced by dislocations, as well as Bi-related defect complexes and defects that emerge similarly in low-temperature grown GaAs [37–39]. Based on the estimated upper limit of the dislocation density in GaAsBi, *ρ* < 1 × 10^8^ cm^−2^, the expected minority carrier lifetime, as reported in [37], would be approximately 0.1 ns, which aligns with our findings. Consequently, further research could focus on reducing the dislocation density in GaAsBi. It is worth noting that increasing the *p*-type carrier concentration to around 5 × 10^16^ cm^−3^ could enhance the *V*_oc_ of the GaAsBi subcell by up to 0.1 V [26]. However, it is important to ensure that doping GaAsBi does not significantly compromise the diffusion length of the carriers. By implementing these optimizations, an efficiency of 30% with a GaAsBi-based triple-junction configuration appears feasible. To achieve efficiency improvements beyond 30%, it would be necessary to reduce the average *W*_oc_ of the 3 J device, minimize optical losses, and mitigate resistance losses [2, 8, 37].

Further research on GaAsBi synthesis remains necessary, particularly focusing on deep-level point-defect control and gaining a better understanding of the nature of defects. Our findings indicate that, in addition to tuning of the III/V ratio guided by the presence of surface Bi and Ga droplet formation, stable growth temperature is a crucial factor in achieving a uniform bismide layer. Additionally, while the step-graded InGaAs buffer layer employed in our study offers one approach to incorporate GaAsBi, future research could explore alternative transparent buffer layers and methods, such as a compositionally graded GaAsBi absorber. The latter would allow for uninterrupted GaAsBi deposition, minimizing surface contamination and potentially enhancing carrier collection efficiency.

## Conclusions

In this study, we presented a thin-film triple-junction solar cell incorporating dilute GaAsBi alloy for the first time. The semiconductor alloy composition of the subcells consisted of AlGaAs/GaAs/GaAsBi with nominally 1.87/1.42/1.0 eV bandgap combination, respectively. The best solar cells exhibited a power conversion efficiency of 19.1%, an open-circuit voltage of 2.51 V, and a short-circuit current of 9.86 mA/cm^2^ under AM1.5G illumination. To grow high-quality 1.0 eV GaAsBi, we used a 340-nm-thick InGaAs buffer layer to expand the lattice constant beyond the GaAs. The bottom subcell employed intrinsic GaAsBi and *p*-on-*n* device polarity, which enhances the extraction of charge carriers generated deeper within the bismide layer. The bismide was 1200 nm thick and took advantage of reflection from the back metal contact to increase the effective optical path. The EQE data and reflectance spectrum suggest that better light management could add 2 mA/cm^2^ to the bismide subcell. Likewise, replacing AlGaAs top subcell with phosphorus-based GaInP would allow for achieving higher overall *V*_oc_. We estimate that the 0.99 eV GaAsBi subcell generated approximately 0.34 V, which translates to a 0.65 V voltage offset, similar to those found in dilute nitrides. The techniques for alloy growth and incorporation of GaAsBi call for further studies to improve its photovoltaic characteristics. This study demonstrates an advanced optoelectronic device based on bismuth-containing alloys and paves the way for their application in high-efficiency solar cells.

## Data Availability

The data that support the findings of this study are available from the corresponding author upon reasonable request.

## References

[1] Yamaguchi M, Dimroth F, Geisz JF (2021). Multi-junction solar cells paving the way for super high-efficiency. J Appl Phys.

[2] Yamaguchi M (2015). Fundamentals and R&D status of III–V compound solar cells and materials. Phys Status Solidi C.

[3] Green MA, Dunlop ED, Hohl-Ebinger J (2022). Solar cell efficiency tables (Version 60). Prog Photovolt Res Appl.

[4] Law DC (2010). Future technology pathways of terrestrial III–V multijunction solar cells for concentrator photovoltaic systems. Sol Energy Mater Sol Cells.

[5] Geisz JF (2007). High-efficiency triple junction solar cells grown inverted with a metamorphic bottom junction. Appl Phys Lett.

[6] http://mldevices.com/index.php/news/. Accessed 10 Feb 2023.

[7] Geisz JF (2020). Six-junction III–V solar cells with 47.1% conversion efficiency under 143 Suns concentration. Nat Energy.

[8] France RM, Geisz JF, Song T, Olavarria W, Young M, Kibbler A, Steiner MA (2022). Triple-junction solar cells with 39.5% terrestrial and 34.2% space efficiency enabled by thick quantum well superlattices. Joule.

[9] Imaizumi M, Takamoto T, Kaneko N, Nozaki Y, Ohshima T (2017). Qualification test results of IMM triple-junction solar cells, space solar sheets, and lightweight and compact solar paddle. E3S Wen Conf.

[10] Paulauskas T (2023). Epitaxial lift-off method for GaAs solar cells with high Al content AlGaAs window layer. Semicond Sci Technol.

[11] Sabnis V, Yuen H, Wiemer M (2012). High-efficiency multijunction solar cells employing dilute nitrides. AIP Conf Proc.

[12] Aho A, Isoaho R, Raappana M (2021). Wide spectral coverage (0.7–2.2 eV) lattice-matched multijunction solar cells based on AlGaInP, AlGaAs and GaInNAsSb materials. Prog Photovolt Res Appl.

[13] Wang S, Lu P (2019). Bismuth-containing alloys and nanostructures.

[14] Alberi K, Dubon OD, Walukiewicz W, Yu KM, Bertulis K, Krotkus A (2007). Valence band anticrossing in GaBi_*x*_As_1−__*x*_. Appl Phys Lett.

[15] Paulauskas T (2022). Epitaxial growth of GaAsBi on thin step-graded InGaAs buffer layers. Semicond Sci Technol.

[16] Paulauskas T (2020). Atomic-resolution EDX, HAADF, and EELS study of GaAs_1__−__x_Bi_x_ alloys. Nanoscale Res Lett.

[17] Paulauskas T (2020). Polarization dependent photoluminescence and optical anisotropy in CuPtB-ordered dilute GaAs_1–__*x*_Bi_*x*_ alloys. J Appl Phys.

[18] Karpus V (2021). Optical anisotropy of CuPt-ordered GaAsBi alloys. J Phys D Appl Phys.

[19] Richards RD (2017). Photovoltaic characterisation of GaAsBi/GaAs multiple quantum well devices. Sol Energy Mater Sol Cells.

[20] Thomas J (2015). Requirements for a GaAsBi 1eV sub-cell in a GaAs-based multi-junction solar cell. Semicond Sci Technol.

[21] Hasegawa S, Kakuyama K, Nishinaka H, Yoshimoto M (2019). PEDOT:PSS/GaAs_1−x_Bi_x_ organic–inorganic solar cells. Jpn J Appl Phys.

[22] Kawata H, Hasegawa S, Nishinaka H, Yoshimoto M (2022). Improving the photovoltaic properties of GaAs/GaAsBi pin diodes by inserting a compositionally graded layer at the hetero-interface. Semicond Sci Technol.

[23] Kakuyama K, Hasegawa S, Nishinaka H (2019). Impact of a small change in growth temperature on the tail states of GaAsBi. J Appl Phys.

[24] Jacobsen H, Puchala B, Kuech TF, Morgan D (2012). Ab initio study of the strain dependent thermodynamics of Bi doping in GaAs. Phys Rev B.

[25] Stevens MA, Grossklaus KA, Vandervelde TE (2019). Strain stabilization of far from equilibrium GaAsBi films. J Cryst Growth.

[26] Paulauskas T (2022). Performance analysis of GaAsBi/InGaAs heterostructure for III–V multi-junction solar cells. Sol Energy Mater Sol Cells.

[27] Meusel M, Baur C, Letay G, Bett AW, Warta W, Fernandez E (2003). Spectral response measurements of monolithic GaInP/Ga(In)As/Ge triple-junction solar cell: measurement artifacts and their explanation. Prog Photovolt Res Appl.

[28] Paulauskas T, Pačebutas V, Geižutis A (2020). GaAs_1__−__x_Bi_x_ growth on Ge: anti-phase domains, ordering, and exciton localization. Sci Rep.

[29] Sugiura H (1988). Double heterostructure GaAs tunnel junction for a AlGaAs/GaAs tandem solar cell. Jpn J Appl Phys.

[30] Miller JN, Collins DM, Moll NJ (1985). Control of Be diffusion in molecular beam epitaxy GaAs. Appl Phys Lett.

[31] Amano C, Sugiura H, Yamaguchi M, Hane K (1989). Fabrication and numerical analysis of AlGaAs/GaAs tandem solar cells with tunnel interconnections. IEEE Trans Electron Dev.

[32] Pettinari G, Patane A, Polimeni A, Capizzi M, Lu X, Tiedje T (2012). Bi-induced p-type conductivity in nominally undoped Ga(AsBi). Appl Phys Lett.

[33] Stevens MA, Lenney S, McElearney J, Grossklaus KA, Vandervelde TE (2020). Characterization of tellurium and silicon as n-type dopants for GaAsBi. Semicond Sci Technol.

[34] Takahashi K, Yamada S, Minagawa Y, Unno T (2001). Characteristics of Al_0.36_Ga_0.64_As/GaAs tandem solar cells with pp−n−n structural AlGaAs solar cells. Sol Energy Mater Sol Cells.

[35] Heckelmann S, Lackner D, Karcher C, Dimroth F, Bett AW (2015). Investigations on Al_x_Ga_1−x_As solar cells grown by MOVPE. IEEE J Photovolt.

[36] Yoshimoto M, Itoh M, Tominaga Y, Oe K (2013). Quantitative estimation of density of Bi-induced localized states in GaAs_1−x_Bi_x_ grown by molecular beam epitaxy. J Cryst Growth.

[37] Yamaguchi M, Dimroth F, Ekins-Daukes NJ, Kojima N, Ohshita Y (2022). Overview and loss analysis of III–V single-junction and multi-junction solar cells. EPJ Photovolt.

[38] Gelczuk Ł, Kopaczek J, Rockett T (2017). Deep-level defects in n-type GaAsBi alloys grown by molecular beam epitaxy at low temperature and their influence on optical properties. Sci Rep.

[39] Fregolent M (2021). Deep levels and carrier capture kinetics in n-GaAsBi alloys investigated by deep level transient spectroscopy. J Phys D Appl Phys.

